# Charge Phenomena in the Elastic Backscattering of Electrons from Insulating Polymers

**DOI:** 10.3390/polym16162329

**Published:** 2024-08-17

**Authors:** Maurizio Dapor

**Affiliations:** 1 European Centre for Theoretical Studies in Nuclear Physics and Related Areas Fondazione Bruno Kessler, 38123 Trento, Italy; dapor@ectstar.eu; 2 Trento Institute for Fundamental Physics and Applications (TIFPA-INFN), 38123 Trento, Italy

**Keywords:** polystyrene, polyethylene, elastic peak electron spectroscopy, charge phenomena, Monte Carlo method

## Abstract

Elastic peak electron spectroscopy (EPES) analyzes the line shape of the elastic peak. The reduction in energy of the elastic peak electrons is the result of energy transfer to the target atoms, a phenomenon known as recoil energy. EPES differs from other electron spectroscopies in its unique ability to identify hydrogen in polymers and hydrogenated carbon-based materials. This feature is particularly noteworthy as lighter elements exhibit stronger energy shifts. The energy difference between the positions of the elastic peak of carbon and the elastic peak of hydrogen tends to increase as the kinetic energy of the incident electrons increases. During electron irradiation of an insulating polymer, if the number of secondary electrons emitted from the surface is less than the number of electrons absorbed in the sample, the surface floats energetically until it stabilizes at a potential energy $eV_{s}$. As a result, the interaction energy changes and modifies the energy difference between the elastic peaks of hydrogen and carbon. In this study, the charge effects are evaluated using the Monte Carlo method to simulate the EPES spectra of electrons interacting with polystyrene and polyethylene.

## 1. Introduction

Recoil energy can be observed in various spectroscopy experiments [1,2]. Its study is particularly valuable because it can be important for the detection of mobile hydrogen in various compounds.

The elastic scattering of electrons from solid targets produces a distinct peak in the electron energy spectra, known as the elastic peak. The line shape analysis of this peak is called elastic peak electron spectroscopy (EPES), also known as electron Compton scattering (ECS) [3,4,5,6,7,8,9,10,11,12]. The intensity of the elastic peak results from the interaction of elastic and inelastic scattering processes. Consequently, EPES is a technique used to determine the inelastic mean free path of electrons, as emphasized, for example, in Refs. [13,14].

It is important to note that the energy transferred to the target atoms reduces the energy of elastically scattered electrons shifting the elastic peak away from the exact center of the beam’s initial kinetic energy. In addition, the recoil effect also leads to broadening (change in peak widths) of the spectra of the elastically backscattered electrons. These effects have been investigated in detail in both experimental and theoretical studies.

The clear meaning of EPES is related to the average recoil energy. It is given by $q^{2}/2M$, where *q* is the transferred momentum and *M* is the mass of the target atom. Because of the 1/M dependence, measuring the energy lost by the electrons in this process can be a useful tool for characterizing the different elements in a compound. On the one hand, instruments with high energy resolution can distinguish the contributions of the different elements. On the other hand, with the relatively low energy resolution usually available in commercial instruments, only the lightest elements that exhibit larger shifts (e.g., hydrogen) can be observed in the elastic peak. Therefore, EPES was proposed as a method to detect hydrogen with commercial instruments, since most other spectroscopy techniques are insensitive to this element.

Applications in other fields are related to the use of similar techniques that are also based on the study of recoil energy, i.e, the same phenomenon on which EPES is based. One important application is the analysis of mobile protons within host structures, which is crucial for the further development of energy technologies. Berger et al. highlighted the use of Elastic Recoil Detection Analysis (ERDA) to study materials by analyzing recoil nuclei driven by ion beams, particularly for profiling light elements such as hydrogen with helium-4 beams [1]. Mayers et al. used neutron Compton scattering (NCS) measurements to determine the kinetic energies of atoms in samples of amorphous hydrogenated carbon, graphite and diamond [15]. Chatzidimitriou et al. measured H and D neutron recoil peaks in time-of-flight (TOF) spectra of H_2_O-D_2_O mixtures and determined the H-to-D cross-section ratio based on their content in the mixture [16].

A very important area for further research is the impact of charge. Materials such as polystyrene and polyethylene, which have dielectric properties, tend to become charged when exposed to electron irradiation. Modeling these charge effects is a major challenge.

Cazaux [17] evaluated the components of the electric field related to the charge distribution induced by electron irradiation in insulators using Maxwell’s equations and taking into account the image effects. In addition, Cazaux introduced the “total yield approach” to study charge effects and determine the sign of charge [18,19]. Joy and colleagues [20,21,22] investigated charge phenomena using low-voltage scanning electron microscopes.

To investigate the measurement of line width using critical dimension scanning electron microscopy, Ciappa et al. [23] and Koschik et al. [24] investigated secondary electron imaging, which modeled adjacent PMMA lines with and without charge effects.

Miotello [25] and Miotello and Dapor [26] developed a model to study the surface electric field of electron-irradiated SiO_2_ targets and its evolution over time. These authors discussed, in particular, the diffusion of the implanted electrons to the surface by ordinary and electric-field-assisted diffusion processes and the recombination with positive charges near the irradiated surface.

As far as the evaluation of the electric field induced on the surface by electron irradiation is concerned, it is possible to measure the surface potential experimentally by observing the energy shift of the secondary electron peak.

The hypothesis of this paper is that charge phenomena influence the energy difference between the elastic peak of carbon and that of hydrogen. While it is true that incident electrons are slowed down by the presence of surface potential, but then accelerated by the same potential as they exit the material—so at first sight, elastically scattered electrons should not be affected by charge phenomena—the landing energy is actually changed, and therefore, the energy difference between the elastic peaks must change. Indeed, a difference in impact energy has a significant effect, especially on the position of the hydrogen peak. In other words, we propose that one way to measure surface potential is based on the fact that the incident electrons are slowed down by the surface potential energy induced by irradiation and the impact energy decreases, so that the energy difference between the elastic hydrogen and carbon peaks also decreases.

## 2. Electron-Induced Charge Phenomena in Insulating Materials

Charge phenomena in insulators, such as those induced by electron irradiation, can be studied theoretically if the absorbed charge and its depth distribution are known. The evaluation of the transport processes of the injected electrons is essential for the calculation of the temporal evolution of the electric field both on the surface and in the depth of the irradiated insulator. Charge diffusion processes generally depend on the electric field, the sample temperature and the electron mobility in the insulator. Electron mobility in particular is a decisive parameter. Another important process is the recombination of charges near the surface, where positive charges remain after the emission of secondary electrons. Since the injected electrons are not simply implanted into the dielectric, but diffuse to the surface by typical diffusion processes supported by the electric field, they can recombine with the positive charges that remain near the irradiated surface after secondary emission. Using a realistic distribution of injected electrons in an insulator, the electric field of the surface of the electron-irradiated solid can be calculated using Gauss’s law. In the continuity equation for the ordinary diffusion process and the diffusion process assisted by an electric field, three terms contribute to the temporal evolution of the space charge: the ordinary diffusion determined by Fick’s first law, the drift velocity of the electrons induced by the electric field and the deposition function of the injected particles. Finally, the boundary condition is set at the surface of the target to account for the recombination of the injected electrons with the positive charges of the surface (i.e., trapped holes) generated by the secondary electron emission. The value of the surface potential $eV_{s}$ and the time required to reach a steady state therefore depend on many parameters, in particular, on the diffusion coefficient, on the number of trapped electrons, on the charge space distribution and on the number of secondary electrons emitted from the region near the surface of the material. In Ref. [26], the time evolution of the electric field at the surface for electrons of a few keV impinging on SiO_2_ was studied by integrating the continuity equation and assuming as the charge source term the depth distribution of the trapped electron obtained by Monte Carlo simulations. The authors showed that the time interval required to reach a steady state is limited to a time interval that is negligible on the time scale typical for the analysis.

According to Joy and Joy, if an insulating material absorbs more electrons than it releases or, conversely, releases more electrons than it absorbs, it becomes electrically charged [22]. The electrical charge influences the energy of the incident and secondary electrons, and in the event of a dielectric breakdown [17], the sample is damaged.

The total emission (secondary electron yield δ plus backscattering coefficient η) is a function of the energy of the primary beam. Due to the initial increase and subsequent decrease in the total electron yield δ+η as a function of the energy of the incident electrons, in a typical situation, there are two values for this energy, $E_{1}$ (between 50 and 150 eV) and $E_{2}$ (between 500 and 3000 eV), for which δ+η=1 applies. At these two energies, dynamic charge equilibrium is established [22,27].

However, if the primary energy of the incident electron beam is higher than $E_{2}$, then the sample becomes negatively charged because the number of secondary electrons emitted from the surface is less than the number of electrons absorbed in the sample, i.e., δ<1−η. According to a simple model proposed by Thornton, the energy of the sample surface continues to float up in energy up to a potential energy $eV_{s}$. So if we use $E_{0}$ to indicate the energy of the incident electrons, the effective landing energy decreases until $E_{0}\;-\;eV_{s}\;=\;E_{2}$, when the electric field of the surface reaches a stationary value [22] (we have indicated the electron charge with *e*).

According to Thornton’s simple model, the value of the stationary potential energy $eV_{s}$ can be calculated as follows [18,27]:

(1) $$e\;V_{s}\;=\;E_{0}-E_{2}\;.$$

The latter equation is actually only valid for

(2) $$e\;I\;R\;\left(1\;-\;\delta \;-\eta \right)\gg \;E_{0}-E_{2}\;,$$

where *I* is the incident beam current [22]. This condition is particularly fulfilled with a high-quality insulator, i.e., if the leakage resistance *R* is very high. If this is not the case, Joy and Joy state that the effective landing energy is greater than $E_{2}$ [22]. Please also note that $E_{2}$ (and thus, $eV_{s}$) varies with the angle of incidence and the surface topography.

## 3. Measurement of Surface Potential $V_{s}$

One possible way to determine $eV_{s}$ experimentally is based on the measurement of the secondary electron peak. If the primary energy of the incident electron beam is higher than $E_{2}$, then the sample becomes negatively charged during electron irradiation. As a result, the electrons are decelerated when approaching the sample and accelerated when leaving the sample. Therefore, $eV_{s}$ can be determined by measuring the shift in the secondary electron peak to higher energies in the spectrum of the outgoing electrons [18].

It seems that the charging of the sample should have no influence on the elastic peak. This is because the incident electrons are decelerated by the surface potential $V_{s}$ when they approach the sample and accelerated by the same potential $V_{s}$ when they leave the sample, which means that the elastic peak does not change. However, this would only be the case if the recoil energy could be neglected. In fact, recoil energy is a phenomenon observed in various spectroscopy experiments. As the incident electrons are slowed down by the surface potential energy $eV_{s}$ induced by the irradiation, the impact energy changes and modifies the energy difference between the elastic peaks of hydrogen and carbon.

## 4. Elastic Peak and Recoil Energy

### 4.1. Classical Theory

The recoil energy $E_{r}$ is given by

(3) $$E_{r}\;=\;\langle E_{r}\rangle \;+\;\Delta \;,$$

where $\langle E_{r}\rangle$ represents the mean recoil energy

(4) $$\langle E_{r}\rangle \;=\;\frac{q^{2}}{2M}\;$$

and *q* is the transferred momentum for a target atom with mass *M*. Δ is the spread in the recoil energy due to atomic vibrations. A Gaussian distribution can therefore be observed, whose standard deviation σ is given by [5,11,28]

(5) $$\sigma \;=\;\sqrt{\frac{4}{3}\langle E_{r}\rangle \langle E_{k}\rangle }\;,$$

where we have specified the mean kinetic energy of the target atoms as $\langle E_{k}\rangle$.

If *E* is the energy of an electron hitting an atom, *m* is the electron mass and θ is the scattering angle, then $\langle E_{r}\rangle$ is given by

(6) $$\langle E_{r}\rangle \;=\;\frac{4m}{M}\;E\;\sin^{2}\frac{\theta }{2}\;.$$

As far as the Monte Carlo method is concerned, we will limit ourselves here to a description of its most important features [12,29,30].

### 4.2. Monte Carlo Simulation

Let us assume spherical coordinates (r,θ,ϕ). An electron beam irradiates the target surface (which lies in the plane z=0) with the primary energy $E_{0}$ and the angle of incidence $\theta_{0}$. The elastic scattering cross-section $\sigma_{\mathrm{el}}$ is calculated by $\sigma_{\mathrm{el}}\;=\;n_{C}\sigma_{C}\;+\;n_{H}\sigma_{H}$. In this equation, $\sigma_{C}$ stands for the elastic scattering cross-sections of carbon, $\sigma_{H}$ for the elastic scattering cross-sections of hydrogen, and $n_{C}$ and $n_{H}$ for their respective atomic concentrations. The inelastic scattering cross-section $\sigma_{\mathrm{inel}}$ is calculated by $\sigma_{\mathrm{inel}}\;=\;{\left(N\lambda_{\mathrm{inel}}\right)}^{-1}$. In this equation, $\lambda_{\mathrm{inel}}$ is the inelastic mean free path of the electrons and *N* is the number of molecules per unit volume. The probabilities for elastic and inelastic scattering are given by $p_{\mathrm{el}}\;=\;\sigma_{\mathrm{el}}/\left(\sigma_{\mathrm{el}}\;+\;\sigma_{\mathrm{inel}}\right)$ and $p_{\mathrm{inel}}\;=\;\sigma_{\mathrm{inel}}/\left(\sigma_{\mathrm{el}}\;+\;\sigma_{\mathrm{inel}}\right)\;=\;1\;-\;p_{\mathrm{el}}$, respectively. After calculating the mean free path of the electrons as $\lambda \;=\;{\left[N\left(\sigma_{\mathrm{el}}\;+\;\sigma_{\mathrm{inel}}\right)\right]}^{-1}$, we can obtain the step length Δs between the collisions using

(7) $$\Delta s=-\lambda \ln(\mu_{1})\;$$

where $\mu_{1}$ is a random number that is sampled with a uniform distribution between 0 and 1. For the choice between elastic and inelastic collisions, a random number $\mu_{2}$ is generated, which is sampled with a uniform distribution between 0 and 1. If $\mu_{2}\;>\;p_{\mathrm{el}}$, the collision is inelastic. In this case, the simulation of the electron’s trajectory is complete, as it is no longer of interest to follow the trajectory of this electron. If, on the other hand, $\mu_{2}\;\leq \;p_{\mathrm{el}}$, then the collision is elastic. If this is the case, another random number $\mu_{3}$ is sampled uniformly between 0 and 1 to determine the type of elastic collision and the recoil energy.

If, on the one hand,

(8) $$0\leq \mu_{3}\leq \frac{n_{C}\sigma_{C}}{\sigma_{\mathrm{el}}}\;,$$

then an electron–carbon collision takes place and the scattering angle θ is determined by

(9) $$\mu_{4}\;=\;P_{C}\left(\theta \right)\;,$$

where $P_{C}\left(\theta \right)$ is the cumulative probability of elastic scattering in C and $\mu_{4}$ is a random number sampled with a uniform distribution between 0 and 1. The azimuth angle ϕ is sampled uniformly between 0 and 2π.

According to Equations (3), (5) and (6), the recoil energy is calculated as follows

(10) $$E_{r}\;=\;\frac{4m}{M_{C}}E\sin^{2}\frac{\theta }{2}\;+\;\Delta_{C}\;.$$

where $\Delta_{C}$ describes the Doppler broadening in C (Δ in Equation (3)).

If, on the other hand,

(11) $$\frac{n_{C}\sigma_{C}}{\sigma_{\mathrm{el}}}<\mu_{3}\leq 1\;,$$

then an electron–hydrogen collision takes place and the scattering angle θ is determined by

(12) $$\mu_{4}\;=\;P_{H}\left(\theta \right)\;,$$

where $P_{H}\left(\theta \right)$ is the cumulative probability of elastic scattering in H and $\mu_{4}$ is a random number sampled with a uniform distribution between 0 and 1. The azimuth angle ϕ is also sampled uniformly between 0 and 2π.

According to Equations (3), (5) and (6), the recoil energy is calculated as follows

(13) $$E_{r}\;=\;\frac{4m}{M_{H}}E\sin^{2}\frac{\theta }{2}\;+\;\Delta_{H}\;.$$

where $\Delta_{H}$ describes the Doppler broadening in H (Δ in Equation (3)).

Since $\Delta_{C}$ and $\Delta_{H}$ are determined using random numbers derived from a Gaussian distribution with the standard deviation calculated according to Equation (5), they can be positive or negative. Please note that $M_{C}$ and $M_{H}$ represent the atomic masses of carbon and hydrogen, respectively. *m* stands for the electron mass and *E* for the electron energy.

The trajectory of each electron is followed until its energy remains greater than a certain threshold value $E_{t}$ and its coordinate *z* (measured from the surface and directed into the interior of the solid) remains greater than zero.

**Figure 1:**
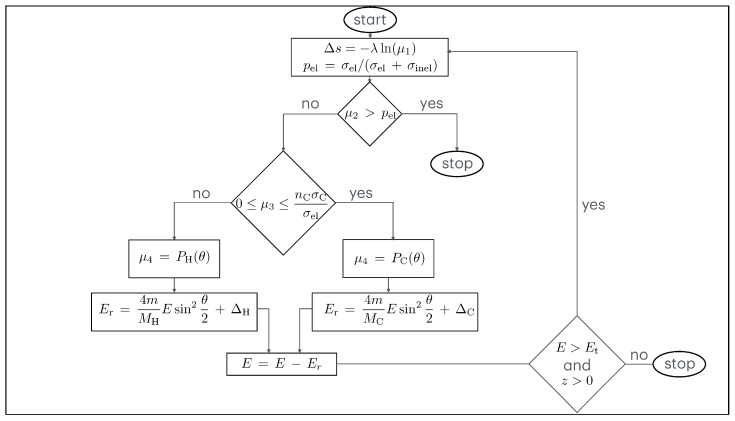
A flow chart of the Monte Carlo simulation. It describes the motion of each electron of the incident beam. The procedure shown here was repeated 10^10^ times to obtain each of the Monte Carlo simulated spectra presented in this paper.

A flow chart showing the Monte Carlo simulation is shown in Figure 1.

## 5. Elastic and Inelastic Scattering Cross-Sections

### 5.1. Elastic Scattering

Both the elastic scattering cross-section and the cumulative probabilities can be obtained once the differential elastic scattering cross-section has been calculated [31,32].

The differential elastic scattering cross-sections of electrons in hydrogen and carbon were calculated using the relativistic partial wave expansion method [12].

**Figure 2:**
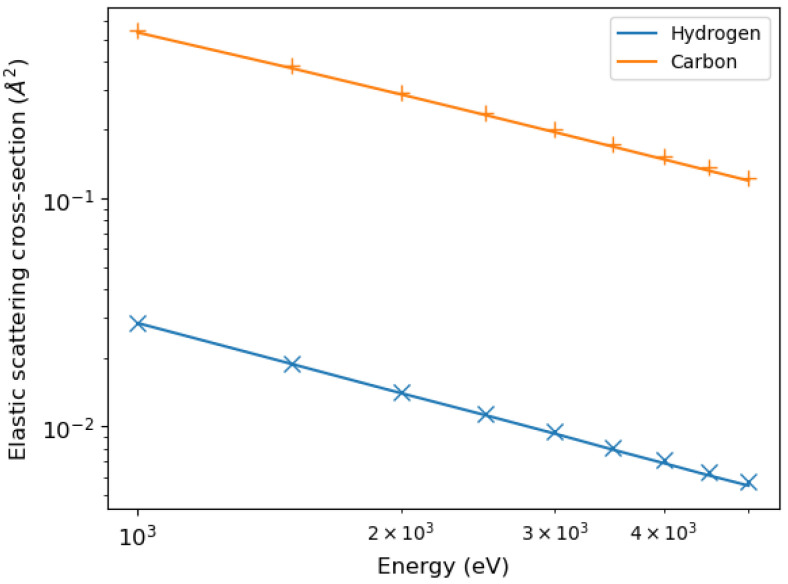
Elastic scattering cross-section of electrons hitting hydrogen and carbon [12] (solid lines) in comparison with the calculations of Mayol and Salvat [31] (symbols).

By integrating the differential elastic scattering cross-sections, we obtained the total elastic scattering cross-sections shown in Figure 2, compared with the calculations of Mayol and Salvat [31].

**Figure 3:**
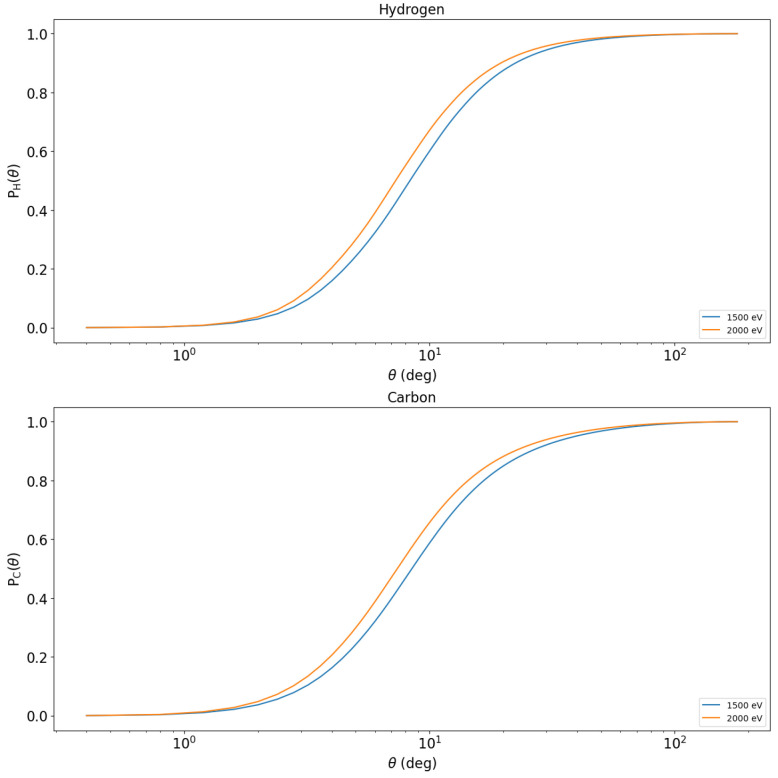
Cumulative probabilities of elastic scattering of 1500 eV and 2000 eV electrons hitting hydrogen and carbon atoms [12].

The integration of the differential elastic scattering cross-sections also allows us to calculate the cumulative probabilities of elastic scattering [12] which are shown in Figure 3. These are monotonically increasing functions that allow us to calculate the scattering angle before each elastic scattering using random variables that are uniformly distributed in the interval between 0 and 1.

### 5.2. Inelastic Scattering

**Figure 4:**
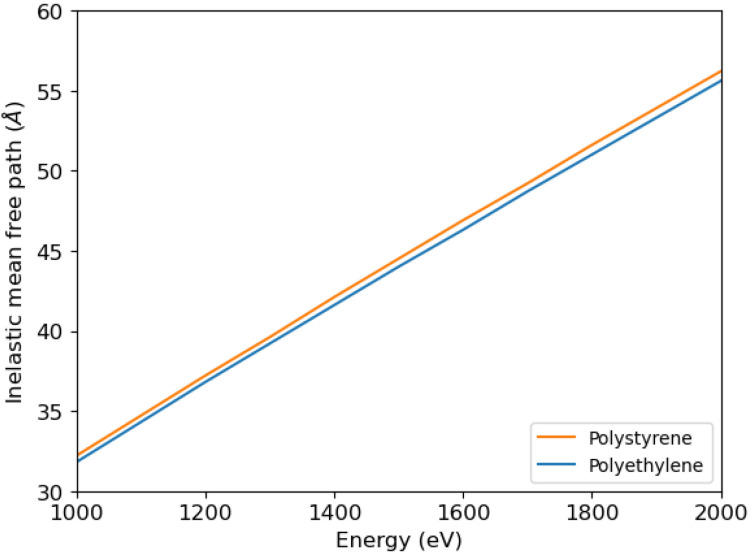
Inelastic mean free path of electrons impinging on polystyrene and polyethylene, according to calculations by Tanuma, Powell, and Penn [34].

The inelastic mean free path can be calculated using Ritchie’s dielectric theory [33], which requires knowledge of the electron energy loss functions. Once the energy loss function is known as a function of energy loss and momentum transfer, the inelastic mean free path can be calculated. In Figure 4 we show the calculations by Tanuma, Powell, and Penn of the inelastic mean free path of electrons impinging on polystyrene and polyethylene [34] used in this article.

## 6. Moving Atoms

The kinetic energy of the vibrating atoms in the solid target depends on the type of atom, the bond, and the temperature. The moving target atoms scatter the distribution of recoil energies, which leads to broadening of the elastic peak known as Doppler broadening [5]. To calculate the standard deviation of the Gaussian distribution describing the Doppler broadening—according to Equation (5)—we used the values of the average kinetic energies of carbon and hydrogen in amorphous hydrogenated carbon reported by Mayers et al. [15], i.e., 103.9 meV for carbon and 145.7 meV for hydrogen.

## 7. Electron-Induced Hydrogen Desorption

As far as the intensity of the hydrogen peak in the EPES spectra is concerned, the damage caused by electron beams is a critical aspect, since hydrogen is desorbed under electron irradiation. The hydrogen content refers to the surface and is lower than the hydrogen content inside the material. In the simulations presented in this paper, we assumed that a small amount of hydrogen was desorbed from the surface by electron irradiation. In particular, 5% hydrogen desorption was assumed in the case of polystyrene [12] and 2.2% hydrogen desorption in the case of polyethylene [13].

## 8. Simulating Elastic Peak Spectra

**Figure 5:**
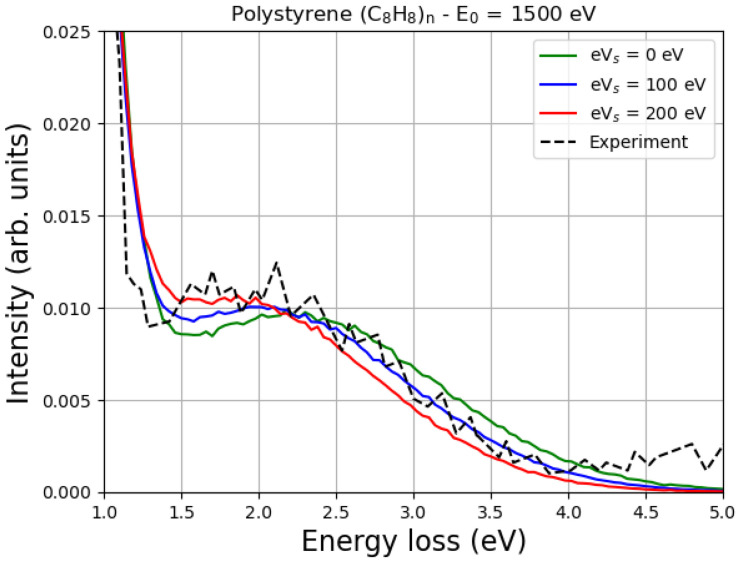
The Monte Carlo simulation of the EPES of 1500 eV electrons impinging on polystyrene, taking charge effects into account. Two values of the surface energy potential $eV_{s}$, i.e., 100 eV and 200 eV, are considered, and the corresponding MC spectra are compared with the spectrum obtained without considering the charge ($eV_{s}=0$ eV). The experimental data of Filippi and Calliari [11] are also presented. The simulations were performed while reproducing the experimental conditions, i.e., the electron beam hit the sample surface at an angle of 30° in the surface normal direction, and the acceptance scattering angle was 138$\;\pm \;6^{\circ }$. The MC simulation was performed assuming 5% hydrogen desorption (induced by the electron irradiation) and taking into account the Doppler broadening. The spectra shown here as a function of energy loss were normalized to a common height of the elastic carbon peak and aligned so that the elastic carbon peak was at 0 energy loss.

Figure 5 shows the Monte Carlo simulation of the EPES of 1500 eV electrons impinging on polystyrene. Two values of the surface energy potential $eV_{s}$, i.e., 100 eV and 200 eV, were considered, and the corresponding MC spectra of the hydrogen elastic peak were compared with the spectrum obtained without considering the charge effects ($eV_{s}$ = 0 eV).

In the same figure, the experimental data of Filippi and Calliari [11] are shown to compare them with the results of our simulations.

According to Joy and Joy [22], the value of $E_{2}$ of polystyrene is 1300 eV, so the application of the simple model represented by Equation (1) for the case of $E_{0}$ = 1500 eV gives $eV_{s}$ = 200 eV.

As already mentioned, typical values of $eV_{s}$ can be smaller than $E_{0}\;-\;E_{2}$ for many reasons. According to a simple model proposed by Joy and Joy [22],

(14) $$eV_{s}\;=\;\frac{eIRf(E_{0}\;-\;E_{2})}{E_{0}\;-\;E_{2}+eIRf}\;,$$

where

(15) f=1−(δ+η),

*I* is the incident beam current, and *R* is the leakage resistance. In addition, $E_{2}$ also depends on the angle of incidence [22].

Consequently, the interval of possible values of $eV_{s}$, for a given primary energy, angle of incidence, incident beam current and material, can range from a few eV (see, e.g., the case of 3 keV electrons impinging on SiO_2_ discussed in detail in [26]) up to several keV [22]. In the case shown in Figure 5 it appears that $eV_{s}$ = 100 eV provides reasonable agreement with the experimental data considered.

**Figure 6:**
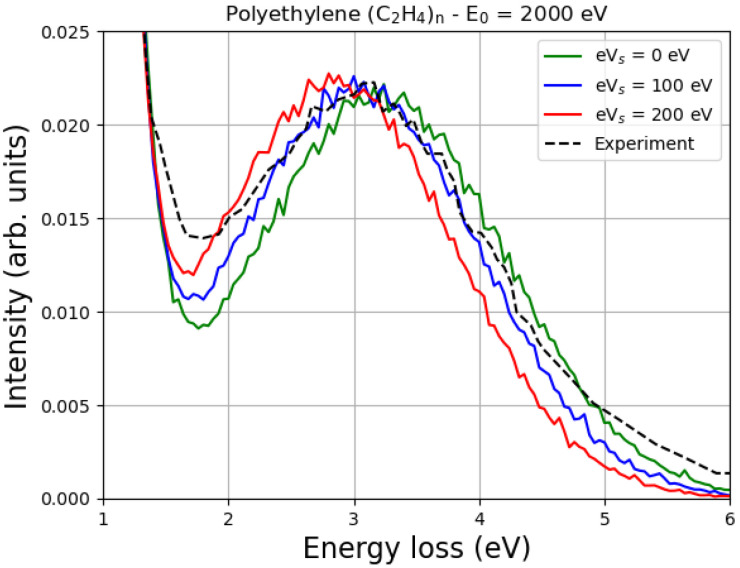
The Monte Carlo simulation of the EPES of 2000 eV electrons impinging on polyethylene, taking charge effects into account. Two values of the surface energy potential $eV_{s}$, i.e., 100 eV and 200 eV, are considered, and the corresponding MC spectra are compared with the spectrum obtained without considering the charge ($eV_{s}=0$ eV). The experimental data of Orosz et al. [13] are also presented. The simulations were performed while reproducing the experimental conditions, i.e., the electron beam hit the sample surface at an angle of 50° in the surface normal direction, and the detection scattering angle was 0° in the surface normal direction. The MC simulation was performed assuming 2.2% hydrogen desorption (induced by the electron irradiation) and taking into account the Doppler broadening. The spectra shown here as a function of energy loss were normalized to a common height of the elastic carbon peak and aligned so that the elastic carbon peak was at 0 energy loss.

The Monte Carlo simulations of the EPES of 2000 eV electrons impinging on polyethylene are shown in Figure 6 and compared with the experimental data of Orosz et al. [13]. Also, in this case, although the value of $E_{2}$ of polyethylene, according to Joy and Joy, is 1500 eV [22], reasonable agreement with the experiment, for this particular sample and these experimental conditions, is achieved with $eV_{s}$ = 100 eV.

It is known that as the primary energy decreases, the energy difference between the carbon elastic peak and the hydrogen elastic peak also decreases. This is quite general, as discussed, for example in Refs. [11,12], and is clearly confirmed in Figure 5 and Figure 6. In particular, please note that at a primary energy of 1500 eV, the hydrogen peak is about 2 eV away from the carbon peak in polystyrene (Figure 5), while at a primary energy of 2000 eV, it is about 3 eV away from the carbon peak in polyethylene (Figure 6). Ignoring the multiple scattering for the moment, this result can be easily understood by looking at Equation (6) and realizing that

(16) $${\langle E_{r}\rangle }_{H}\;-\;{\langle E_{r}\rangle }_{C}\;=\;4m\;E\;\sin^{2}\frac{\theta }{2}\left[\frac{1}{M_{H}}-\frac{1}{M_{C}}\right]\;.$$

where we have indicated the mean recoil energy of hydrogen as ${\langle E_{r}\rangle }_{H}$ and the mean recoil energy of carbon as ${\langle E_{r}\rangle }_{C}$. Multiple scattering, which is accurately described by the Monte Carlo simulation, confirms that the energy difference between the carbon elastic peak and the hydrogen elastic peak decreases as the energy of the incident electrons decreases.

In particular, an increase in $eV_{s}$ means a decrease in the landing energy, i.e., the energy of the impact, which is smaller than the primary energy due to the charge effects. This means that as $eV_{s}$ increases, the difference between the carbon elastic peak and the hydrogen elastic peak must decrease, as shown in Figure 5 and Figure 6.

It can also be seen from Figure 5 and Figure 6 that the values of the surface energy potential of 0 eV, 100 eV and 200 eV do not have such a great influence on the shape of the peak. The reason for this is that, as already discussed, in a first approximation, the elastic peak should not be affected very much. In fact, incident electrons are decelerated by the surface potential when they enter the sample and accelerated by the same potential when they exit the material. However, as the landing energy changes, the energy difference between the elastic peaks must also change, as clearly shown in Figure 5 and Figure 6.

The implications of these simulations are manifold. First of all, it is quite clear that when working with insulating polymers, the effects of charging cannot be ignored. These effects can, in principle, be measured by the position of the hydrogen elastic peak relative to the position of the carbon elastic peak. Furthermore, if high-resolution analytical instruments are available, the line width of the elastic peaks allows the average kinetic energy of the hydrogen and carbon atoms in the polymer to be determined. Finally, it is also evident that this technique allows a quantitative analysis of the presence of hydrogen on the material surface.

The applications of these simulations in industry and research are numerous, especially in connection with the analysis and characterization of polymers. It is obvious that this Monte Carlo modeling can of course be extended to polymers containing oxygen and other chemical elements in addition to carbon and hydrogen. In any case, it should not be forgotten that EPES is the most suitable of the electronic spectroscopies for detecting and quantifying the presence of hydrogen. Moreover, this technique can also be used to perform more fundamental studies on radiation damage, as it is indeed able to detect and quantify the desorption of hydrogen from the surface caused by irradiation.

## 9. Critical Aspects

Please note that this Monte Carlo simulation assumes homogeneity of the material. The anisotropy of the target affects the elastic scattering cross-sections. In order to account for the effects of anisotropy on these cross-sections, it is necessary to consider multiple scattering interactions with neighboring molecules. This approach is described in detail, e.g., in Ref. [35], for the calculation of the elastic scattering cross-section of electrons hitting liquid water.

Another major challenge is the low intensity of the hydrogen signal compared to the signals of the other elements. The cross-section of the elastic scattering increases with the square of the atomic number, so that the signal intensity increases sharply at higher atomic numbers. As shown in Ref. [11], this complicates the detection of hydrogen embedded in materials with a high atomic number and the identification of low hydrogen concentrations in general.

## 10. Contribution to the Theoretical Understanding of Electron Backscattering and Charge Phenomena

The results of this research and, in general, modeling with the Monte Carlo method are very useful for the theoretical understanding of electron backscattering and charging phenomena, since experimental results often need to be interpreted. In the absence of a theoretical approach, such as the one proposed here, the mere observation of the spectrum does not allow a complete assessment of the various features of the spectrum, such as (i) the energy position of the carbon and hydrogen elastic peak maxima (which are associated with the recoil energies and have been accurately determined by Monte Carlo modeling of multiple collisions in the solid state), (ii) the full width at half maximum (due to a combination of the Doppler effect, analyzer resolution, and electron source energy distribution), and (iii) the signal intensities (which depend on the hydrogen and carbon content as well as the hydrogen desorption induced by electron irradiation).

## 11. Conclusions

After a description of recoil phenomena and their potential applications in materials science (e.g., the detection of hydrogen in carbon-based materials, the analysis of mobile protons within host structures—crucial for the further development of energy technologies—and the evaluation of the average kinetic energy of hydrogen in polymers), this article provides a brief summary of classical recoil theory and the method used for simulating the spectra (Monte Carlo). Insulating polymers were considered to discuss the effects of surface charging after electron irradiation. In particular, simulations of the EPES spectra of keV electrons interacting with polystyrene and polyethylene were presented and compared with experimental data. In the Monte Carlo calculations, Doppler broadening was taken into account to describe the elastic collision with moving carbon and hydrogen atoms. In addition, electron-induced hydrogen desorption was taken into account. This method made it possible to evaluate the potential surface energy.

The difference between the energy of the elastic peak of carbon (the main elastic peak) and that of the elastic peak of hydrogen is an increasing function of the incident electron beam primary energy. In this paper, for example, it is shown that this energy difference is approximately 2 eV when the primary energy of the incident electron beam is 1500 eV, and 3 eV when the primary energy of the incident electron beam is 2000 eV.

Since the surface of an insulating sample is electrically charged by the irradiation, an electric field is generated on the surface, which slows down the incident electrons. The landing energy is therefore lower than the nominal primary energy, so the energy difference between the elastic peaks of carbon and hydrogen must also be lower than expected if the surface potential energy were not present. In this sense, measuring the position of the hydrogen elastic peak in relation to the carbon elastic peak and comparing it with the values obtained using the Monte Carlo method for different values of surface potential energy allows the intensity of this potential to be evaluated. In the two cases studied in this work, for example, this comparison showed that the best agreement with the experiment was obtained assuming that the surface potential energy was in the order of 100 eV.

In summary, the EPES technique is not only useful for the assessment of hydrogen content in carbon-based materials (an assessment that can be performed by measuring the ratio between the areas of the hydrogen and carbon elastic peaks), but can also be used effectively for the quantitative determination of the surface potential caused by the electron irradiation of dielectric polymers.

## Data Availability

All data supporting the results of this study are included in the article.
